# Genome Sequence of *Lactobacillus sakei* LK-145 Isolated from a Japanese Sake Cellar as a High Producer of d-Amino Acids

**DOI:** 10.1128/genomeA.00656-17

**Published:** 2017-08-17

**Authors:** Shiro Kato, Tadao Oikawa

**Affiliations:** a High Technology Research Core, Kansai University, Suita, Osaka, Japan; b Faculty of Chemistry, Materials, and Bioengineering, Kansai University, Suita, Osaka, Japan

## Abstract

This announcement reports the complete genome sequence of strain LK-145 of *Lactobacillus sakei* isolated from a Japanese sake cellar as a potent strain for the production of large amounts of d-amino acids. Three putative genes encoding an amino acid racemase were identified.

## GENOME ANNOUNCEMENT

Important physiological events in mammals, such as neurotransmission (1), testosterone synthesis (2), and fertilization (3), are known to involve d-amino acids, including d-Asp. These d-amino acids are considered to be synthesized endogenously and/or are derived from enteric bacteria. Recent analyses suggest that not only endogenous d-amino acids but also exogenous ones might have physiological functions in eukaryotic bodies. For instance, oral administration of dl-Asp with feed increases the motility of rabbit spermatozoa (4), and drinking d-Asp solution alleviates the symptoms of neuropathy in mice (5).

*Lactobacillus sakei* strain LK-145 was isolated from a Japanese sake cellar and has the potential to produce large amounts of three d-amino acids, d-Ala, d-Glu, and d-Asp (6). *L. sakei* is one of the major bacteria in the traditional sake-brewing process, called “kimoto,” and sake contains a high concentration of d-amino acids, including d-Asp (7). This high d-amino acid producer might be useful for applications in the production of d-amino acid-enriched fermentative foods. To obtain the genetic basis for understanding the mechanism of high d-amino acid production in *L. sakei* strain LK-145, we performed whole-genome analysis of strain LK-145.

The complete genome sequencing of strain LK-145 was performed using a GS Junior 454 platform (Roche). A whole-genome shotgun library and an 8-kb-span paired-end library were constructed using a GS Titanium rapid library preparation kit and GS Titanium library paired-end adaptors. Assembly of the whole-genome shotgun reads and paired-end reads and the remaining gap filling were carried out using GS De Novo Assembler version 2.9 and the Sanger sequencing method, respectively.

The genome of strain LK-145 consists of one chromosome of 1,950,487 bp and three plasmids of 33,266, 6,196, and 4,315 bp. Genome coverage was approximately 70-fold, and the G+C contents of the chromosome and the three plasmids were 41.23, 40.01, 35.93, and 35.34%, respectively. Prediction and annotation of the coding sequences using the Microbial Genome Annotation Pipeline (8) indicated that the chromosome contained 1,981 genes encoding proteins, 65 genes for tRNAs, and 15 genes for rRNAs. Three plasmids contained 37, 7, and 5 genes probably encoding proteins, respectively. All genes predicted to encode proteins related to amino acid metabolism were located on the chromosome. The putative amino acid metabolic pathways constructed using the KEGG Automatic Annotation Server (9) were quite similar to those of the lactic acid bacterium *L. sakei* 23K isolated from fresh sausage (10), which is the first genome sequenced strain of *L. sakei*, and 3 putative amino acid racemase genes (Ala, Asp, and Glu racemases) were identified on the chromosome of strain LK-145 as well as in strain 23K. Although no remarkable differences in the genes predicted to catalyze amino acid metabolism, including the d-amino acid synthesis reaction, were detected, the conservation of some significant metabolic pathways, such as the lactate metabolic pathway, was different in the two strains.

### Accession number(s).

The complete genome sequence has been deposited in DDBJ under the GenBank accession numbers AP017931 (chromosome), AP017932 (plasmid), AP017933 (plasmid) and AP017934 (plasmid).
